# A hydrogen-bridged adduct 3,4,6,7,8,9-hexa­hydro-2*H*-pyrimido[1,2-*a*]pyrimidin-1-ium [1,3-bis­(*tert*-butyl­dimethyl­sil­yloxy)-1,3-bis­(pyridin-2-yl)propan-2-yl­idene]nitro­nate acetonitrile monosolvate

**DOI:** 10.1107/S1600536811033927

**Published:** 2011-08-27

**Authors:** Martin Schulz, Helmar Görls, Matthias Westerhausen

**Affiliations:** a Institut für Anorganische und Analytische Chemie, Friedrich-Schiller-Universität, Jena, Humboldt-Strasse 8, 07743 Jena, Germany

## Abstract

The title compound, C_7_H_14_N_3_^+^·C_25_H_40_N_3_O_4_Si_2_^−^·CH_3_CN, was obtained by the reaction of 2-nitro-1,3-di(pyridin-2-yl)-1,3-di(*tert*-butyl­dimethyl­sil­yloxy)propane with 1,3,4,6,7,8-hexa­hydro-2*H*-pyrimido[1,2-*a*]pyrimidine. Two hydrogen bonds stabilize the Lewis acid/base pair of the nitro­nate and the guanidinium moiety with N⋯O distances of 2.772 (3) and 2.732 (3) Å. Both hydrogen atoms are more closely bound to the guanidinium [N—H distances of 0.83 (3) and 0.93 (3) Å] than to the nitro­nate moiety. The nitro­nate is double-bonded to the respective carbon with an N=C bond length of 1.316 (3) Å.

## Related literature

For the synthesis of 2-nitro-1,3-di(pyridin-2-yl)-1,3-di(*tert*-butyl­dimethyl­sil­yloxy)propane, see: Schulz *et al.* (2011 ▶). For Nef reactions (conversion of nitro compounds into carbonyl compounds) with amidines or guanidines, see: Ballini *et al.* (2002 ▶). For a general review of Nef reactions, see: Ballini & Petrini (2004 ▶). For a comparison of bond lengths, see: Allen *et al.* (1987 ▶).
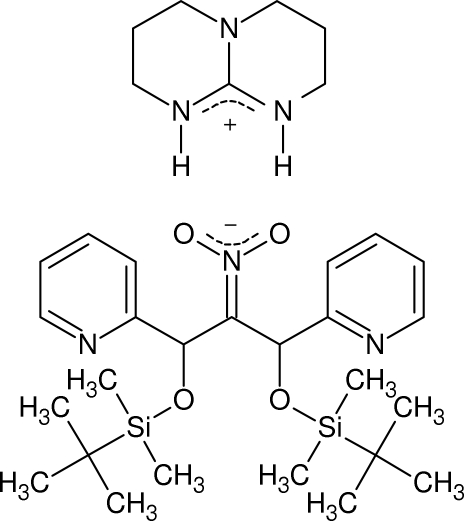


## Experimental

### 

#### Crystal data

C_7_H_14_N_3_^+^·C_25_H_40_N_3_O_4_Si_2_^−^·CH_3_CN
*M* *_r_* = 684.05
Triclinic, 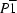

*a* = 9.4335 (8) Å
*b* = 11.1149 (9) Å
*c* = 19.4529 (14) Å
α = 103.062 (4)°
β = 98.098 (4)°
γ = 95.197 (5)°
*V* = 1951.2 (3) Å^3^
*Z* = 2
Mo *K*α radiation
μ = 0.14 mm^−1^
*T* = 183 K
0.06 × 0.06 × 0.04 mm

#### Data collection

KappaCCD diffractometer
12006 measured reflections
8497 independent reflections
3820 reflections with *I* > 2σ(*I*)
*R*_int_ = 0.048

#### Refinement

*R*[*F* ^2^ > 2σ(*F* ^2^)] = 0.058
*wR*(*F* ^2^) = 0.141
*S* = 0.92
8497 reflections
442 parameters
H atoms treated by a mixture of independent and constrained refinement
Δρ_max_ = 0.25 e Å^−3^
Δρ_min_ = −0.27 e Å^−3^

### 

Data collection: *COLLECT* (Nonius, 1998 ▶); cell refinement: *DENZO* (Otwinowski & Minor 1997 ▶); data reduction: *DENZO*; program(s) used to solve structure: *SHELXTL* (Sheldrick, 2008 ▶); program(s) used to refine structure: *SHELXTL*; molecular graphics: *SHELXTL*; software used to prepare material for publication: *SHELXTL*.

## Supplementary Material

Crystal structure: contains datablock(s) I, global. DOI: 10.1107/S1600536811033927/nk2105sup1.cif

Structure factors: contains datablock(s) I. DOI: 10.1107/S1600536811033927/nk2105Isup2.hkl

Supplementary material file. DOI: 10.1107/S1600536811033927/nk2105Isup3.mol

Supplementary material file. DOI: 10.1107/S1600536811033927/nk2105Isup4.cml

Additional supplementary materials:  crystallographic information; 3D view; checkCIF report

## Figures and Tables

**Table 1:** Hydrogen-bond geometry (Å, °)

*D*—H⋯*A*	*D*—H	H⋯*A*	*D*⋯*A*	*D*—H⋯*A*
N4—H1*N*4⋯O3	0.83 (3)	1.94 (3)	2.772 (3)	172 (3)
N6—H1*N*6⋯O4	0.93 (3)	1.80 (3)	2.732 (3)	178 (3)

**Fig. 1.**
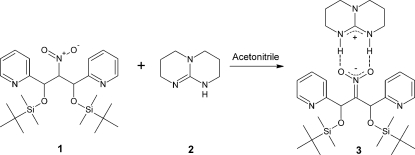
The synthetic pathway to 3.

**Fig. 2.**
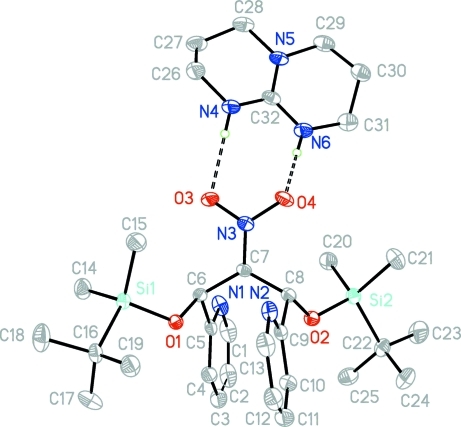
Molecular structure of the title compound 3; displacement ellipsoids are at the 40% probability level. Solvent acetonitrile and H atoms are omitted.

## supplementary crystallographic information

### Comment

Secondary aliphatic nitrocompounds can be transformed into the respective ketone with amidine or guanidine bases (Ballini *et al.* 2002, Ballini *et al.* 2004). This procedure represents a valuable route to synthetically important ketones, especially in the presence of functionalities, which are sensitive towards oxidants, reductants or acids. Compound 1 was previously described and the title compound 3 was found as a white precipitate from the reaction of 1 with 2 in acetonitrile (Schulz *et al.* (2011)). The title compound 3 represents an adduct between a nitronate anion and a guanidinium cation held together by two hydrogen bonds between the nitronate oxygen atoms and the guanidinium nitrogen atoms. The donor-donor distances (N4—O3 2.772 (3) Å and N6—O4 2.732 (3) Å) indicate a strong interaction. Both nitronate N—O bond lengths are similar (N3—O3 1.312 (3) Å and N3—O4 1.319 (3) Å) with values intermediate between a single and a double bond. The nitronate C7=N3 bond length (1.316 (3) Å) indicates a double bond, and this conclusion is supported by an angle sum of 359.84° for C7. The guanidinium cation shows the typical C—N bond lengths. All other bond lengths and angles are within the expected range.(Allen *et al.* 1987)

### Experimental

Compound 1 was prepared according to the procedure given in (Schulz *et al.* (2011)) and compound 2 was commercially obtained. 1 (0.20 g, 0.4 mmol) and 2 (0.12 g, 0.4 mmol) were dissolved in 1.5 ml of acetonitrile and stirred at room temperature. The solution turned yellow immediately and after 10 min the title compound 3 crystallized as a colorless solid. Subsequently, the crystalline product was collected by filtration yielding 0.10 g (31%).

### Refinement

N-bound H atoms were located by difference Fourier synthesis and freely refined. The refined N-H distances are 0.83 (3) Å for N(4)-H(1N4) and 0.93 (3) Å for N(6)-H(1N6) respectively. All other hydrogen atoms were set to idealized positions and were refined with 1.2 times (1.5 for methyl groups) the isotropic displacement parameter of the corresponding carbon atom. The methyl groups were allowed to rotate but not to tip.

### Crystal data

**Table:**

C_7_H_14_N_3_^+^·C_25_H_40_N_3_O_4_Si_2_^−^·CH_3_CN	*Z* = 2
*M**_r_* = 684.05	*F*(000) = 740
Triclinic, *P*1	*D*_x_ = 1.164 Mg m^−^^3^
Hall symbol: -P 1	Mo *K*α radiation, λ = 0.71073 Å
*a* = 9.4335 (8) Å	Cell parameters from 12006 reflections
*b* = 11.1149 (9) Å	θ = 1.9–27.5°
*c* = 19.4529 (14) Å	µ = 0.14 mm^−^^1^
α = 103.062 (4)°	*T* = 183 K
β = 98.098 (4)°	Prism, colourless
γ = 95.197 (5)°	0.06 × 0.06 × 0.04 mm
*V* = 1951.2 (3) Å^3^	

### Data collection

**Table:**

KappaCCD diffractometer	3820 reflections with *I* > 2σ(*I*)
Radiation source: fine-focus sealed tube	*R*_int_ = 0.048
graphite	θ_max_ = 27.5°, θ_min_ = 1.9°
φ and ω scan	*h* = −12→11
12006 measured reflections	*k* = −14→13
8497 independent reflections	*l* = −25→25

### Refinement

**Table:**

Refinement on *F*^2^	Primary atom site location: structure-invariant direct methods
Least-squares matrix: full	Secondary atom site location: difference Fourier map
*R*[*F*^2^ > 2σ(*F*^2^)] = 0.058	Hydrogen site location: inferred from neighbouring sites
*wR*(*F*^2^) = 0.141	H atoms treated by a mixture of independent and constrained refinement
*S* = 0.92	*w* = 1/[σ^2^(*F*_o_^2^) + (0.0529*P*)^2^] where *P* = (*F*_o_^2^ + 2*F*_c_^2^)/3
8497 reflections	(Δ/σ)_max_ = 0.001
442 parameters	Δρ_max_ = 0.25 e Å^−^^3^
0 restraints	Δρ_min_ = −0.27 e Å^−^^3^

### Special details

**Table:**

Geometry. All e.s.d.'s (except the e.s.d. in the dihedral angle between two l.s. planes) are estimated using the full covariance matrix. The cell e.s.d.'s are taken into account individually in the estimation of e.s.d.'s in distances, angles and torsion angles; correlations between e.s.d.'s in cell parameters are only used when they are defined by crystal symmetry. An approximate (isotropic) treatment of cell e.s.d.'s is used for estimating e.s.d.'s involving l.s. planes.
Refinement. Refinement of *F*^2^ against ALL reflections. The weighted *R*-factor *wR* and goodness of fit *S* are based on *F*^2^, conventional *R*-factors *R* are based on *F*, with *F* set to zero for negative *F*^2^. The threshold expression of *F*^2^ > σ(*F*^2^) is used only for calculating *R*-factors (gt) *etc*. and is not relevant to the choice of reflections for refinement. *R*-factors based on *F*^2^ are statistically about twice as large as those based on *F*, and *R*- factors based on ALL data will be even larger.

### Fractional atomic coordinates and isotropic or equivalent isotropic displacement parameters (Å^2^)

**Table:**

	*x*	*y*	*z*	*U*_iso_*/*U*_eq_	
Si1	0.28003 (8)	0.83299 (8)	0.16632 (4)	0.0308 (2)	
Si2	−0.25909 (9)	0.49919 (8)	0.28617 (4)	0.0333 (2)	
O1	0.15141 (19)	0.72021 (17)	0.16400 (8)	0.0291 (5)	
O2	−0.18244 (19)	0.55522 (17)	0.22672 (9)	0.0315 (5)	
O3	0.1918 (2)	0.81398 (19)	0.36422 (9)	0.0404 (5)	
O4	−0.0422 (2)	0.81554 (19)	0.36965 (9)	0.0411 (6)	
N1	0.1652 (3)	0.4769 (2)	0.25806 (13)	0.0444 (7)	
N2	−0.1677 (2)	0.8364 (2)	0.16444 (12)	0.0360 (6)	
N3	0.0581 (3)	0.7801 (2)	0.33126 (11)	0.0325 (6)	
C1	0.1591 (4)	0.3524 (4)	0.2417 (2)	0.0578 (10)	
H1A	0.1823	0.3130	0.2796	0.069*	
C2	0.1212 (4)	0.2784 (3)	0.1734 (2)	0.0640 (11)	
H2A	0.1166	0.1904	0.1646	0.077*	
C3	0.0904 (4)	0.3351 (3)	0.1186 (2)	0.0562 (10)	
H3A	0.0647	0.2868	0.0706	0.067*	
C4	0.0971 (3)	0.4633 (3)	0.13363 (16)	0.0422 (8)	
H4A	0.0773	0.5044	0.0962	0.051*	
C5	0.1330 (3)	0.5307 (3)	0.20375 (14)	0.0314 (7)	
C6	0.1453 (3)	0.6715 (3)	0.22614 (13)	0.0270 (7)	
H6A	0.2382	0.7025	0.2599	0.032*	
C7	0.0243 (3)	0.7157 (2)	0.26432 (13)	0.0260 (7)	
C8	−0.1330 (3)	0.6834 (3)	0.23320 (13)	0.0288 (7)	
H8A	−0.1873	0.7338	0.2675	0.035*	
C9	−0.1740 (3)	0.7144 (3)	0.16117 (13)	0.0296 (7)	
C10	−0.2241 (3)	0.6228 (3)	0.09868 (14)	0.0380 (8)	
H10A	−0.2260	0.5371	0.0983	0.046*	
C11	−0.2711 (3)	0.6599 (4)	0.03691 (16)	0.0477 (9)	
H11A	−0.3056	0.5998	−0.0068	0.057*	
C12	−0.2673 (3)	0.7845 (4)	0.03977 (17)	0.0501 (9)	
H12A	−0.3006	0.8123	−0.0017	0.060*	
C13	−0.2142 (3)	0.8685 (3)	0.10378 (17)	0.0441 (8)	
H13A	−0.2103	0.9547	0.1050	0.053*	
C14	0.4593 (3)	0.7866 (3)	0.19464 (16)	0.0518 (9)	
H14A	0.4682	0.7808	0.2446	0.078*	
H14B	0.5356	0.8490	0.1902	0.078*	
H14C	0.4687	0.7055	0.1641	0.078*	
C15	0.2491 (4)	0.9748 (3)	0.23070 (16)	0.0540 (10)	
H15A	0.2582	0.9599	0.2788	0.081*	
H15B	0.1519	0.9952	0.2168	0.081*	
H15C	0.3207	1.0443	0.2306	0.081*	
C16	0.2656 (3)	0.8527 (3)	0.07238 (14)	0.0350 (7)	
C17	0.2813 (4)	0.7296 (3)	0.02151 (15)	0.0590 (10)	
H17A	0.2779	0.7413	−0.0271	0.088*	
H17B	0.2023	0.6663	0.0220	0.088*	
H17C	0.3739	0.7022	0.0370	0.088*	
C18	0.3861 (4)	0.9523 (4)	0.06847 (19)	0.0620 (10)	
H18A	0.3810	0.9597	0.0190	0.093*	
H18B	0.4802	0.9283	0.0848	0.093*	
H18C	0.3741	1.0325	0.0992	0.093*	
C19	0.1206 (3)	0.8948 (3)	0.04847 (15)	0.0486 (9)	
H19A	0.1159	0.9053	−0.0004	0.073*	
H19B	0.1109	0.9742	0.0807	0.073*	
H19C	0.0419	0.8318	0.0498	0.073*	
C20	−0.1201 (3)	0.4739 (3)	0.35725 (15)	0.0481 (9)	
H20A	−0.0645	0.5539	0.3834	0.072*	
H20B	−0.0552	0.4182	0.3355	0.072*	
H20C	−0.1673	0.4361	0.3905	0.072*	
C21	−0.3779 (4)	0.6056 (3)	0.33040 (16)	0.0529 (9)	
H21A	−0.3236	0.6886	0.3504	0.079*	
H21B	−0.4120	0.5734	0.3689	0.079*	
H21C	−0.4609	0.6110	0.2952	0.079*	
C22	−0.3662 (3)	0.3496 (3)	0.23180 (16)	0.0407 (8)	
C23	−0.4412 (4)	0.2833 (3)	0.28070 (19)	0.0689 (11)	
H23A	−0.4944	0.2038	0.2523	0.103*	
H23B	−0.5084	0.3358	0.3030	0.103*	
H23C	−0.3685	0.2682	0.3180	0.103*	
C24	−0.4825 (3)	0.3756 (3)	0.17450 (17)	0.0600 (10)	
H24A	−0.5383	0.2968	0.1466	0.090*	
H24B	−0.4357	0.4170	0.1426	0.090*	
H24C	−0.5471	0.4296	0.1980	0.090*	
C25	−0.2680 (4)	0.2638 (3)	0.19317 (17)	0.0537 (9)	
H25A	−0.3266	0.1879	0.1632	0.081*	
H25B	−0.1969	0.2419	0.2286	0.081*	
H25C	−0.2179	0.3071	0.1631	0.081*	
N4	0.2139 (3)	0.9307 (3)	0.50788 (14)	0.0444 (7)	
N5	0.1491 (3)	0.9190 (2)	0.61673 (11)	0.0392 (7)	
N6	0.0099 (3)	0.8051 (3)	0.50982 (14)	0.0418 (7)	
C26	0.3300 (4)	1.0306 (3)	0.54181 (16)	0.0527 (9)	
H26A	0.2915	1.1117	0.5511	0.063*	
H26B	0.4032	1.0338	0.5102	0.063*	
C27	0.3971 (3)	1.0049 (3)	0.61136 (15)	0.0548 (10)	
H27A	0.4757	1.0722	0.6365	0.066*	
H27B	0.4386	0.9252	0.6018	0.066*	
C28	0.2810 (3)	0.9979 (3)	0.65717 (15)	0.0485 (9)	
H28A	0.3180	0.9642	0.6982	0.058*	
H28B	0.2579	1.0829	0.6764	0.058*	
C29	0.0369 (4)	0.8974 (3)	0.65895 (15)	0.0492 (9)	
H29A	0.0336	0.9748	0.6954	0.059*	
H29B	0.0613	0.8321	0.6842	0.059*	
C30	−0.1094 (4)	0.8573 (3)	0.61245 (15)	0.0484 (9)	
H30A	−0.1461	0.9296	0.5973	0.058*	
H30B	−0.1784	0.8249	0.6399	0.058*	
C31	−0.0971 (4)	0.7569 (3)	0.54713 (15)	0.0481 (9)	
H31A	−0.0674	0.6820	0.5619	0.058*	
H31B	−0.1916	0.7331	0.5151	0.058*	
C32	0.1243 (4)	0.8866 (3)	0.54528 (15)	0.0380 (8)	
C1AN	−0.5773 (5)	0.6466 (4)	0.4939 (2)	0.0603 (10)	
N1AN	−0.4736 (4)	0.6891 (4)	0.5320 (2)	0.0942 (13)	
C2AN	−0.7089 (4)	0.5903 (4)	0.44558 (18)	0.0683 (11)	
H2A1	−0.7914	0.6036	0.4707	0.102*	
H2A2	−0.7055	0.5008	0.4286	0.102*	
H2A3	−0.7192	0.6284	0.4047	0.102*	
H1N4	0.204 (3)	0.901 (3)	0.4638 (16)	0.056 (10)*	
H1N6	−0.007 (4)	0.810 (3)	0.4622 (18)	0.074 (12)*	

### Atomic displacement parameters (Å^2^)

**Table:**

	*U*^11^	*U*^22^	*U*^33^	*U*^12^	*U*^13^	*U*^23^
Si1	0.0295 (5)	0.0311 (5)	0.0322 (4)	0.0002 (4)	0.0076 (4)	0.0086 (4)
Si2	0.0347 (5)	0.0350 (5)	0.0333 (4)	0.0039 (4)	0.0088 (4)	0.0125 (4)
O1	0.0322 (11)	0.0313 (12)	0.0242 (9)	−0.0030 (9)	0.0057 (8)	0.0097 (8)
O2	0.0400 (12)	0.0235 (11)	0.0305 (10)	−0.0029 (9)	0.0091 (9)	0.0067 (8)
O3	0.0369 (13)	0.0497 (14)	0.0270 (10)	0.0021 (11)	−0.0029 (9)	−0.0003 (10)
O4	0.0444 (13)	0.0528 (14)	0.0257 (10)	0.0167 (11)	0.0093 (9)	0.0029 (10)
N1	0.0491 (17)	0.0402 (18)	0.0563 (16)	0.0162 (14)	0.0234 (14)	0.0237 (14)
N2	0.0319 (14)	0.0387 (17)	0.0441 (14)	0.0093 (13)	0.0126 (12)	0.0178 (12)
N3	0.0382 (16)	0.0344 (15)	0.0259 (12)	0.0072 (13)	0.0066 (12)	0.0074 (11)
C1	0.057 (2)	0.050 (3)	0.086 (3)	0.022 (2)	0.031 (2)	0.037 (2)
C2	0.065 (3)	0.027 (2)	0.109 (3)	0.0078 (19)	0.043 (3)	0.014 (2)
C3	0.054 (2)	0.035 (2)	0.072 (2)	−0.0049 (18)	0.025 (2)	−0.0088 (19)
C4	0.045 (2)	0.035 (2)	0.0443 (18)	−0.0015 (16)	0.0165 (16)	0.0023 (16)
C5	0.0291 (17)	0.0323 (18)	0.0365 (16)	0.0025 (14)	0.0147 (14)	0.0108 (14)
C6	0.0285 (16)	0.0309 (18)	0.0219 (14)	0.0009 (14)	0.0025 (12)	0.0093 (12)
C7	0.0335 (17)	0.0240 (16)	0.0216 (14)	0.0040 (13)	0.0062 (12)	0.0064 (12)
C8	0.0337 (17)	0.0277 (18)	0.0261 (14)	0.0040 (14)	0.0097 (13)	0.0057 (13)
C9	0.0247 (16)	0.037 (2)	0.0303 (15)	0.0055 (14)	0.0088 (13)	0.0122 (14)
C10	0.0391 (18)	0.044 (2)	0.0307 (16)	0.0040 (16)	0.0071 (14)	0.0081 (15)
C11	0.041 (2)	0.070 (3)	0.0325 (17)	0.0064 (19)	0.0035 (15)	0.0138 (17)
C12	0.035 (2)	0.082 (3)	0.043 (2)	0.018 (2)	0.0075 (16)	0.031 (2)
C13	0.039 (2)	0.050 (2)	0.057 (2)	0.0173 (17)	0.0149 (17)	0.0301 (18)
C14	0.037 (2)	0.074 (3)	0.0483 (19)	0.0051 (19)	0.0053 (16)	0.0241 (18)
C15	0.068 (2)	0.037 (2)	0.054 (2)	−0.0059 (19)	0.0232 (18)	0.0021 (17)
C16	0.0286 (17)	0.042 (2)	0.0399 (17)	0.0033 (15)	0.0111 (14)	0.0189 (15)
C17	0.079 (3)	0.068 (3)	0.0347 (17)	0.023 (2)	0.0213 (18)	0.0094 (17)
C18	0.044 (2)	0.078 (3)	0.076 (2)	−0.003 (2)	0.0122 (19)	0.046 (2)
C19	0.040 (2)	0.064 (2)	0.0456 (18)	0.0090 (18)	0.0063 (15)	0.0205 (17)
C20	0.050 (2)	0.058 (2)	0.0393 (17)	0.0073 (18)	0.0073 (16)	0.0190 (16)
C21	0.059 (2)	0.056 (2)	0.054 (2)	0.0141 (19)	0.0268 (18)	0.0206 (18)
C22	0.0390 (19)	0.036 (2)	0.0484 (18)	−0.0039 (16)	0.0052 (16)	0.0176 (16)
C23	0.071 (3)	0.056 (3)	0.080 (3)	−0.017 (2)	0.016 (2)	0.025 (2)
C24	0.045 (2)	0.060 (3)	0.068 (2)	−0.0090 (19)	−0.0033 (19)	0.015 (2)
C25	0.060 (2)	0.041 (2)	0.054 (2)	0.0020 (19)	0.0021 (18)	0.0050 (17)
N4	0.0462 (18)	0.0513 (19)	0.0281 (14)	0.0029 (15)	0.0030 (14)	−0.0029 (14)
N5	0.0502 (17)	0.0420 (17)	0.0238 (13)	0.0143 (14)	0.0027 (12)	0.0035 (12)
N6	0.0517 (18)	0.0429 (18)	0.0277 (14)	0.0035 (15)	0.0027 (14)	0.0056 (13)
C26	0.045 (2)	0.065 (3)	0.0428 (19)	0.005 (2)	0.0051 (17)	0.0057 (18)
C27	0.047 (2)	0.062 (3)	0.0430 (19)	0.0157 (19)	−0.0073 (16)	−0.0066 (17)
C28	0.058 (2)	0.048 (2)	0.0341 (17)	0.0195 (19)	−0.0021 (16)	0.0007 (16)
C29	0.073 (3)	0.048 (2)	0.0300 (16)	0.0137 (19)	0.0096 (18)	0.0129 (16)
C30	0.065 (2)	0.049 (2)	0.0371 (17)	0.0108 (19)	0.0170 (17)	0.0168 (16)
C31	0.061 (2)	0.047 (2)	0.0390 (17)	0.0090 (19)	0.0049 (17)	0.0176 (16)
C32	0.046 (2)	0.039 (2)	0.0300 (17)	0.0201 (17)	0.0057 (16)	0.0047 (15)
C1AN	0.061 (3)	0.056 (3)	0.063 (3)	0.009 (2)	0.018 (2)	0.008 (2)
N1AN	0.068 (3)	0.105 (3)	0.092 (3)	0.005 (2)	0.010 (2)	−0.007 (2)
C2AN	0.066 (3)	0.072 (3)	0.068 (2)	0.013 (2)	0.006 (2)	0.022 (2)

### Geometric parameters (Å, °)

**Table:**

Si1—O1	1.652 (2)	C18—H18C	0.9800
Si1—C15	1.853 (3)	C19—H19A	0.9800
Si1—C14	1.858 (3)	C19—H19B	0.9800
Si1—C16	1.877 (3)	C19—H19C	0.9800
Si2—O2	1.6516 (18)	C20—H20A	0.9800
Si2—C20	1.855 (3)	C20—H20B	0.9800
Si2—C21	1.857 (3)	C20—H20C	0.9800
Si2—C22	1.872 (3)	C21—H21A	0.9800
O1—C6	1.438 (3)	C21—H21B	0.9800
O2—C8	1.431 (3)	C21—H21C	0.9800
O3—N3	1.312 (3)	C22—C25	1.537 (4)
O4—N3	1.319 (3)	C22—C23	1.536 (4)
N1—C5	1.339 (3)	C22—C24	1.546 (4)
N1—C1	1.342 (4)	C23—H23A	0.9800
N2—C13	1.336 (3)	C23—H23B	0.9800
N2—C9	1.339 (4)	C23—H23C	0.9800
N3—C7	1.316 (3)	C24—H24A	0.9800
C1—C2	1.374 (5)	C24—H24B	0.9800
C1—H1A	0.9500	C24—H24C	0.9800
C2—C3	1.367 (5)	C25—H25A	0.9800
C2—H2A	0.9500	C25—H25B	0.9800
C3—C4	1.382 (4)	C25—H25C	0.9800
C3—H3A	0.9500	N4—C32	1.320 (4)
C4—C5	1.378 (4)	N4—C26	1.454 (4)
C4—H4A	0.9500	N4—H1N4	0.83 (3)
C5—C6	1.516 (4)	N5—C32	1.335 (3)
C6—C7	1.507 (3)	N5—C29	1.463 (4)
C6—H6A	1.0000	N5—C28	1.467 (4)
C7—C8	1.500 (4)	N6—C32	1.339 (4)
C8—C9	1.523 (4)	N6—C31	1.455 (4)
C8—H8A	1.0000	N6—H1N6	0.93 (3)
C9—C10	1.389 (4)	C26—C27	1.510 (4)
C10—C11	1.385 (4)	C26—H26A	0.9900
C10—H10A	0.9500	C26—H26B	0.9900
C11—C12	1.371 (5)	C27—C28	1.513 (4)
C11—H11A	0.9500	C27—H27A	0.9900
C12—C13	1.374 (4)	C27—H27B	0.9900
C12—H12A	0.9500	C28—H28A	0.9900
C13—H13A	0.9500	C28—H28B	0.9900
C14—H14A	0.9800	C29—C30	1.509 (4)
C14—H14B	0.9800	C29—H29A	0.9900
C14—H14C	0.9800	C29—H29B	0.9900
C15—H15A	0.9800	C30—C31	1.516 (4)
C15—H15B	0.9800	C30—H30A	0.9900
C15—H15C	0.9800	C30—H30B	0.9900
C16—C17	1.529 (4)	C31—H31A	0.9900
C16—C19	1.531 (4)	C31—H31B	0.9900
C16—C18	1.536 (4)	C1AN—N1AN	1.133 (5)
C17—H17A	0.9800	C1AN—C2AN	1.442 (5)
C17—H17B	0.9800	C2AN—H2A1	0.9800
C17—H17C	0.9800	C2AN—H2A2	0.9800
C18—H18A	0.9800	C2AN—H2A3	0.9800
C18—H18B	0.9800		
			
O1—Si1—C15	108.18 (12)	H19A—C19—H19B	109.5
O1—Si1—C14	109.79 (13)	C16—C19—H19C	109.5
C15—Si1—C14	110.35 (15)	H19A—C19—H19C	109.5
O1—Si1—C16	105.85 (12)	H19B—C19—H19C	109.5
C15—Si1—C16	112.70 (14)	Si2—C20—H20A	109.5
C14—Si1—C16	109.84 (13)	Si2—C20—H20B	109.5
O2—Si2—C20	110.59 (13)	H20A—C20—H20B	109.5
O2—Si2—C21	112.33 (13)	Si2—C20—H20C	109.5
C20—Si2—C21	107.53 (14)	H20A—C20—H20C	109.5
O2—Si2—C22	103.46 (11)	H20B—C20—H20C	109.5
C20—Si2—C22	112.02 (15)	Si2—C21—H21A	109.5
C21—Si2—C22	110.96 (15)	Si2—C21—H21B	109.5
C6—O1—Si1	119.55 (16)	H21A—C21—H21B	109.5
C8—O2—Si2	125.65 (15)	Si2—C21—H21C	109.5
C5—N1—C1	117.1 (3)	H21A—C21—H21C	109.5
C13—N2—C9	116.7 (3)	H21B—C21—H21C	109.5
O3—N3—C7	123.0 (2)	C25—C22—C23	109.5 (3)
O3—N3—O4	115.57 (19)	C25—C22—C24	108.0 (3)
C7—N3—O4	121.5 (2)	C23—C22—C24	108.8 (3)
N1—C1—C2	124.0 (3)	C25—C22—Si2	110.7 (2)
N1—C1—H1A	118.0	C23—C22—Si2	109.7 (2)
C2—C1—H1A	118.0	C24—C22—Si2	110.1 (2)
C3—C2—C1	118.1 (3)	C22—C23—H23A	109.5
C3—C2—H2A	121.0	C22—C23—H23B	109.5
C1—C2—H2A	121.0	H23A—C23—H23B	109.5
C2—C3—C4	119.3 (3)	C22—C23—H23C	109.5
C2—C3—H3A	120.4	H23A—C23—H23C	109.5
C4—C3—H3A	120.4	H23B—C23—H23C	109.5
C5—C4—C3	119.1 (3)	C22—C24—H24A	109.5
C5—C4—H4A	120.5	C22—C24—H24B	109.5
C3—C4—H4A	120.5	H24A—C24—H24B	109.5
N1—C5—C4	122.5 (3)	C22—C24—H24C	109.5
N1—C5—C6	114.1 (2)	H24A—C24—H24C	109.5
C4—C5—C6	123.4 (3)	H24B—C24—H24C	109.5
O1—C6—C7	111.7 (2)	C22—C25—H25A	109.5
O1—C6—C5	108.84 (19)	C22—C25—H25B	109.5
C7—C6—C5	112.2 (2)	H25A—C25—H25B	109.5
O1—C6—H6A	108.0	C22—C25—H25C	109.5
C7—C6—H6A	108.0	H25A—C25—H25C	109.5
C5—C6—H6A	108.0	H25B—C25—H25C	109.5
N3—C7—C8	117.4 (2)	C32—N4—C26	121.4 (3)
N3—C7—C6	117.9 (2)	C32—N4—H1N4	120 (2)
C8—C7—C6	124.6 (2)	C26—N4—H1N4	119 (2)
O2—C8—C7	111.9 (2)	C32—N5—C29	122.0 (3)
O2—C8—C9	109.0 (2)	C32—N5—C28	121.8 (3)
C7—C8—C9	114.3 (2)	C29—N5—C28	115.3 (2)
O2—C8—H8A	107.1	C32—N6—C31	121.5 (3)
C7—C8—H8A	107.1	C32—N6—H1N6	110 (2)
C9—C8—H8A	107.1	C31—N6—H1N6	125 (2)
N2—C9—C10	123.3 (3)	N4—C26—C27	107.7 (3)
N2—C9—C8	114.3 (2)	N4—C26—H26A	110.2
C10—C9—C8	122.2 (3)	C27—C26—H26A	110.2
C11—C10—C9	118.2 (3)	N4—C26—H26B	110.2
C11—C10—H10A	120.9	C27—C26—H26B	110.2
C9—C10—H10A	120.9	H26A—C26—H26B	108.5
C12—C11—C10	119.1 (3)	C26—C27—C28	108.4 (3)
C12—C11—H11A	120.5	C26—C27—H27A	110.0
C10—C11—H11A	120.5	C28—C27—H27A	110.0
C13—C12—C11	118.6 (3)	C26—C27—H27B	110.0
C13—C12—H12A	120.7	C28—C27—H27B	110.0
C11—C12—H12A	120.7	H27A—C27—H27B	108.4
N2—C13—C12	124.0 (3)	N5—C28—C27	112.0 (2)
N2—C13—H13A	118.0	N5—C28—H28A	109.2
C12—C13—H13A	118.0	C27—C28—H28A	109.2
Si1—C14—H14A	109.5	N5—C28—H28B	109.2
Si1—C14—H14B	109.5	C27—C28—H28B	109.2
H14A—C14—H14B	109.5	H28A—C28—H28B	107.9
Si1—C14—H14C	109.5	N5—C29—C30	111.5 (2)
H14A—C14—H14C	109.5	N5—C29—H29A	109.3
H14B—C14—H14C	109.5	C30—C29—H29A	109.3
Si1—C15—H15A	109.5	N5—C29—H29B	109.3
Si1—C15—H15B	109.5	C30—C29—H29B	109.3
H15A—C15—H15B	109.5	H29A—C29—H29B	108.0
Si1—C15—H15C	109.5	C29—C30—C31	109.5 (3)
H15A—C15—H15C	109.5	C29—C30—H30A	109.8
H15B—C15—H15C	109.5	C31—C30—H30A	109.8
C17—C16—C19	109.4 (3)	C29—C30—H30B	109.8
C17—C16—C18	108.9 (2)	C31—C30—H30B	109.8
C19—C16—C18	108.0 (3)	H30A—C30—H30B	108.2
C17—C16—Si1	109.8 (2)	N6—C31—C30	108.6 (3)
C19—C16—Si1	110.93 (18)	N6—C31—H31A	110.0
C18—C16—Si1	109.7 (2)	C30—C31—H31A	110.0
C16—C17—H17A	109.5	N6—C31—H31B	110.0
C16—C17—H17B	109.5	C30—C31—H31B	110.0
H17A—C17—H17B	109.5	H31A—C31—H31B	108.4
C16—C17—H17C	109.5	N4—C32—N5	121.1 (3)
H17A—C17—H17C	109.5	N4—C32—N6	118.3 (3)
H17B—C17—H17C	109.5	N5—C32—N6	120.5 (3)
C16—C18—H18A	109.5	N1AN—C1AN—C2AN	178.9 (5)
C16—C18—H18B	109.5	C1AN—C2AN—H2A1	109.5
H18A—C18—H18B	109.5	C1AN—C2AN—H2A2	109.5
C16—C18—H18C	109.5	H2A1—C2AN—H2A2	109.5
H18A—C18—H18C	109.5	C1AN—C2AN—H2A3	109.5
H18B—C18—H18C	109.5	H2A1—C2AN—H2A3	109.5
C16—C19—H19A	109.5	H2A2—C2AN—H2A3	109.5
C16—C19—H19B	109.5		

### Hydrogen-bond geometry (Å, °)

**Table:**

*D*—H···*A*	*D*—H	H···*A*	*D*···*A*	*D*—H···*A*
N4—H1N4···O3	0.83 (3)	1.94 (3)	2.772 (3)	172 (3)
N6—H1N6···O4	0.93 (3)	1.80 (3)	2.732 (3)	178 (3)
